# Updated check-list of the mayflies (Insecta: Ephemeroptera) of Iraq

**DOI:** 10.3897/BDJ.9.e63830

**Published:** 2021-03-25

**Authors:** Farhad A. Khudhur, Pavel Sroka

**Affiliations:** 1 University of Sulaimani, Sulaymaniyah, Kurdistan Region, Iraq University of Sulaimani Sulaymaniyah, Kurdistan Region Iraq; 2 Biology Centre of the Czech Academy of Sciences, Institute of Entomology, České Budějovice, Czech Republic Biology Centre of the Czech Academy of Sciences, Institute of Entomology České Budějovice Czech Republic

**Keywords:** aquatic biodiversity, mayflies, Ephemeroptera, Middle East, Iraq

## Abstract

Based on a recent field survey in Iraqi Kurdistan and a critical evaluation of previously published data, 37 mayfly species are listed as occurring in Iraq. We collected and identified nine species as new for the country and corrected some previously published records. For several species scarcely treated in the literature, we provide information allowing their identification in the larval stage to promote the acquisition of reliable faunistic data from Iraq in the future.

## Introduction

Faunistic studies of mayflies occurring in some parts of the Middle East are still sparse. Existing studies have mainly focused on the Arabian Peninsula (Thomas and Sartori 1989, Sartori and Gillies 1990, [Bibr B6531906][Bibr B6533783]), Levant (Demoulin 1973, Koch 1980, Koch 1981, Koch 1988, Thomas et al. 2007, Thomas et al. 1988, Thomas and Dia 1983, Thomas and Dia 1984, Thomas and Dia 1985, Thomas and Dia 1999, Thomas and Dia 1982, Sartori 1992, Gattolliat et al. 2012, Malzacher 1992) and Iran (Bojková et al. 2018, Staniczek et al. 2020, Hrivniak et al. 2020). Turkey has also been extensively studied (e.g. Kazancı and Türkmen 2012, Kazancı and Türkmen 2016, Salur et al. 2016, Sroka et al. 2019a, Hrivniak et al. 2019). In contrast to abovementioned regions, Iraq has been studied only poorly (Al-Zubaidi et al. 1987, Bojková and Soldán 2015, Abdul-Rassoul 2020).

The stream network of Iraq includes the Tigris River basin which has several sub-basins (Khabur, Greater Zab, Lesser Zab, Adhaim and Diyala) and the Euphrates River basin (Al-Ansari et al. 2019). More than fifty watercourses flow from Turkey or Iran into Iraq (Yousuf et al. 2018). The discharge of both the Tigris and Euphrates is decreasing with time due to the construction of dams in the upstream part of the basins and climate change (Al-Gburi et al. 2017, Al-Ansari et al. 2018). In addition, the increasing stress of wastewater effluents has led to the degradation of many habitats, for example, in the southern wetlands and marshes (Al-Gburi et al. 2017). Mayfly larvae are well known for their sensitivity to oxygen depletion, therefore, they are commonly used as a suitable model in freshwater monitoring programmes (Bauernfeind and Moog 2000, Menetrey et al. 2008). However, at present, there are not enough data for Iraq to analyse these processes and a good knowledge of the local fauna is a necessary prerequisite for such efforts.

Most of the published data concerning the mayflies of Iraq are restricted to the northern region of the country. Al-Zubaidi and Al-Kayatt (1986) presented information on the geographical distribution of five families, six genera and five newly-recorded species from northern Iraq. Then, Al-Zubaidi et al. (1987) published nine mayfly species as newly recorded for Iraq and described two new species, *Oligoneuriella bicaudata* Al-Zubaidi, Braasch & Al-Kayatt, 1987 and *Isonychia arabica* Al-Zubaidi, Braasch & Al-Kayatt, 1987. Four newly-recorded species from the middle region of Iraq were recorded by Carl (1989). Another new species, *Prosopistoma helenae* Bojková & Soldán, 2015 was discovered from the Tigris River in Mosul city. A remarkable mayfly species, *Mortogenesia mesopotamica* (Morton, 1921), has been thoroughly re-described by Soldán and Godunko (2013). Several other papers on the mayfly diversity of Iraq have been published quite recently and represent ecological studies focused on aquatic macroinvertebrates that are useful in water quality bioassessment (Shekha 2011, Hanna and Shekha 2015, Ali and Latef 2017 and Hanna et al. 2019). The first checklist of the mayfly fauna of Iraq was summarised by Abdul-Rassoul (2020), based on literature data only. This checklist compiled 30 species in 18 genera and 10 families; however, some specimens were actually identified only on the genus level.

Most of the studies dealing with Iraqi fauna have been undertaken using the morphological approach only. The barcoding fragment of mtDNA (COI) was acquired for some locally-collected species by M. Al Saffar, but remains mostly unpublished (some of these sequences were incorporated in Sroka et al. 2019). Recently, Khudhur and Shekha (2020) have performed a molecular species identification (using 16S rDNA) of some Heptageniidae taxa.

Recent ecological studies or attempts to incorporate molecular data into mayfly research in Iraq are potentially very useful. They are, however, also hampered by a lack of suitable literature allowing accurate species identifications. Therefore, the samples are often identified using inappropriate literature sources, which might result in considerable confusion regarding faunistic data in the future (see Braasch (1980)). Therefore, we aim to partly provide the necessary information to avoid this eventuality. Specifically, our study aims to: i) provide a list of the mayfly species occurring in Iraq, based on all published records to date and to critically evaluate these records; ii) based on our sampling, report the finding of a further nine species previously unknown from the country; iii) provide essential information about the diagnostic characters of some scarcely reported species to facilitate their identification and the future assembly of sound faunistic data from the country.

## Material and methods

**Figure 1.**
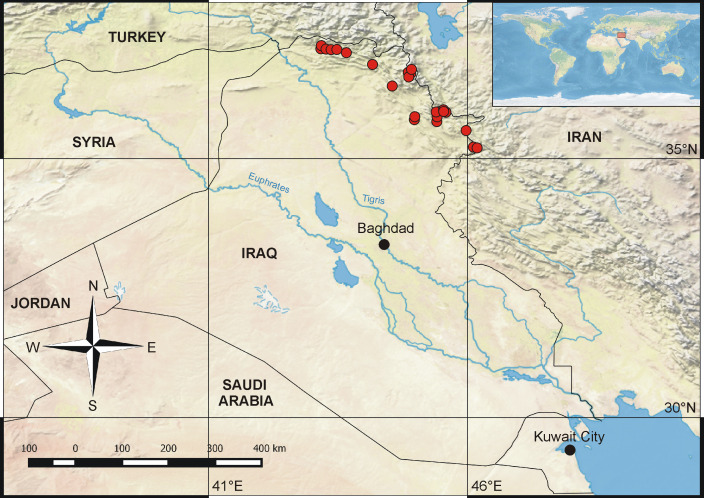
Map of Iraq, localities sampled marked by red dots.

The samples were collected semi-quantitatively by a D-frame net using the kick-sampling method, sweeping through roots and submerged plants or picked manually from rocks and pebbles. All of the material was collected by F. Khudhur from January to October 2019. The samples were collected from 24 sites (one sample per site) which were selected to cover the mountainous region of the northern part of the country. For the list of localities, see Table 1. The larvae were stored in 96% EtOH at -20°C. The material totals 667 specimens and is housed at the Biology Centre CAS, Institute of Entomology (IECA). A map of all the localities sampled is provided in Fig. 1. To check minute morphological characters, parts of some specimens were mounted on microscopic slides using HydroMatrix (MicroTech Lab, Graz, Austria) mounting medium (soluble in water). Photographs were obtained using a Canon EOS 1200D camera mounted on a Leica M205 C stereomicroscope. All photographs were subsequently enhanced with Adobe Photoshop™ CS5.

## Results

### Check-list of Mayflies in Iraq

Literature published up to now contains records of 37 species occurring in Iraq. Table 2 summarises all of the records (excluding eight additional species listed in Table 3, which were certainly reported, based on incorrect identification, see Discussion). Only taxa identified to the species-level are included. We report nine species as new for the fauna of Iraq. We collected fresh material only in the northern part of the country. The southern part of Iraq hosts a very different species composition, since the environment is totally different. Its further study would be highly beneficial, but at present, it is hampered by the difficult accessibility of these locations due to the unstable security situation. Thus, for the fauna of S. Iraq in the current check-list, we relied on the literature data only.

### Diagnostic characters of selected species

Here, we list and illustrate the most pronounced diagnostic characters of several species (including species we report as new for the country) to facilitate accurate identification and to prevent the publication of confusing faunistic and ecological data, especially for non-taxonomists. Most of these species are not included in available identification keys, focusing on European fauna (e.g. Studemann et al. 1992, Engblom 1996,Bauernfeind and Humpesch 2001, Eiseler 2005).

**Baetis (Rhodobaetis) braaschi Zimmermann, 1980**

**Figure 2.**
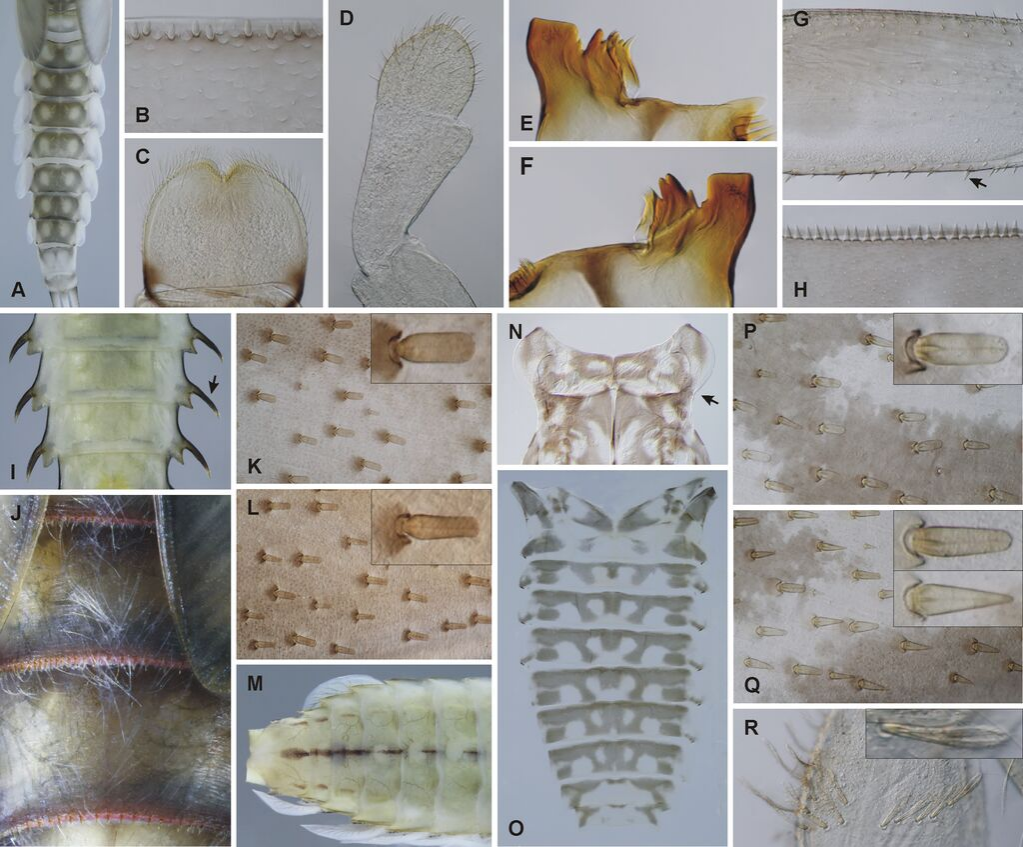
Determination characters of selected species: **A.** *Baetis braaschi*, colouration of abdominal terga; **B.** *Baetis ilex*, posterior margin of abdominal terga; **C.** *B. ilex*, labrum; **D.** *B. ilex*, labial palp; **E.** *Baetis samochai*, apical part of left mandible; **F.** *B. samochai*, apical part of right mandible; **G.** *B. samochai*, middle section of femur with short setae on posterior margin (arrow); **H.** *B. samochai*, posterior margin of abdominal terga; **I.** *Epeorus zaitzevi*, section of abdomen ventrally with lateral protuberances (arrow); **J.** *E. zaitzevi*, details of abdominal terga; **K.** *Ecdyonurus ornatipennis*, setae on basal part of hind femur; **L.** *E. ornatipennis*, setae on distal part of hind femur; **M.** *E. ornatipennis*, colouration of abdominal sterna; **N.** *Electrogena pseudaffinis*, part of thorax with rounded posterior corners of pronotum (arrow); **O.** *E. pseudaffinis*, colouration of abdominal terga; **P.** *E. pseudaffinis*, setae on basal part of middle femur; **Q.** *E. pseudaffinis*, setae on distal part of middle femur; **R.** *Caenis luctuosa*, setae on forefemur.

The species belongs to the subgenus Rhodobaetis Jacob, 2003. Its subgeneric placement can be readily confirmed by the presence of a row of articulated setae (spatulas) on the posterior margin of the abdominal terga. It mostly lacks spine-like setae on the margins of the gill plates, thus being easily recognisable from *B. rhodani* and *B. ilex*, other *Rhodobaetis* species frequently co-occurring in the region and always equipped with such setae. Furthermore, *B. braaschi* is distinct from all other *Baetis* species in the region by the presence of two rounded pale spots on the abdominal terga (Fig. 2A). The species is not covered in European keys, its morphology being treated in Godunko et al. (2004) or Sroka et al. (2012). We suspect that *B. braaschi* is sometimes misidentified as *Baetis vernus* Curtis, 1834 (reported for Iraq by Shekha 2011), the presence of which in the Middle East we consider doubtful. *Baetis vernus* is a common European species with somewhat similar markings on the abdominal terga. This misidentification can be easily avoided by checking the presence of setae on the posterior margins of the abdominal terga, indicative of *Rhodobaetis*.

**Baetis (Rhodobaetis) ilex Jacob & Zimmermann, 1978**

The species represents another member of the subgenus Rhodobaetis, easily distinguishable by the presence of setae on the posterior margins of the abdominal terga (Fig. 2B) and spine-like setae on the gill margins. It can be distinguished from all other *Rhodobaetis* species of the Caucasus and Middle East by: i) the presence of spine-like setae on both the inner and outer margins of the gill plates, although these on the inner margin tend to be scarce in specimens from Iraq. The most common *Rhodobaetis* species, *B. rhodani*, exhibits these setae on the outer margin only; ii) the elongated shape of the labrum (Fig. 2C) and relatively narrow labial palps (Fig. 2D); iii) the absence of triangular projections on the posterior margin of the abdominal terga (Fig. 2B). The illustrations in the original description of the species (Jacob and Zimmermann 1978) are of very good quality and can be reliably used for species identification.

**Baetis (Baetis) samochai Koch, 1981**

The species is distinct from all other *Baetis* species occurring in the region in several characters: i) a very broad outer tooth of the mandibular incisors, distinctly separated from the inner part of the incisors (Fig. 2E, F); ii) short and pointed setae on the posterior margin of the femora (Fig. 2G); iii) narrow pointed spines on the posterior margin of the terga (Fig. 2H). An overview of *B. samochai* morphology including some illustrations, based on material from Israel, is also available in Yanai et al. (2018).

**Epeorus (Epeorus) zaitzevi Tshernova, 1981**

Despite the species being relatively widespread and common in the region, existing descriptions are scattered and sometimes confusing. In the original description, Tshernova (1981) described only male imago. Braasch 1978 described a larva of *E. zaitzevi*, but erroneously under the name *Epeorus znojkoi*, as pointed out by Koch (1988) and Sartori (1992). Some information on *E. zaitzevi* larvae is also contained in Samocha (1972) and Demoulin (1973), where the species is treated as *Epeorus* sp. It is also included in the key of Kluge (1997).

*E. zaitzevi* larva is similar to European *Epeorus assimilis* Eaton, 1883-88, sharing the absence of paracercus and not having an enlarged gill I. Nevertheless, it can be distinguished by the following combination of characters: i) long and pointed posterolateral projections on the abdominal terga (Fig. 2I); ii) the presence of long, hair-like setae on the abdominal terga (Fig. 2J); iii) long, sparse teeth on the posterior margin of the abdominal terga. Sartori (1992) mentioned the presence of a narrow dark band on the posterior margin of each abdominal tergite, visible in both larvae and adults, clearly visible also on our material.

**Ecdyonurus (Ecdyonurus) ornatipennis Tshernova, 1938**

The morphology of this species is poorly known. In the original description, Tshernova (1938) studied only the male imago. The only description of larva was published by Braasch (1980), although he noted the association of his larval material with Tshernova's adults as questionable, based on the proximity of localities and not direct rearing. Nevertheless, the Braasch's concept of *E. ornatipennis* larvae was followed by subsequent authors (Kazancı and Braasch (1988), Hrivniak et al. (2018)) and we follow it here as well, as a thorough revision of Caucasian *Ecdyonurus* is not yet available. From all other species of *Ecdyonurus* occurring in the area, *E. ornatipennis* can be distinguished by: i) posterolateral projections of the prothorax which are short and apically not pointed; ii) setae on the dorsal surface of hind femora which are apically not pointed (Fig. 2K, L); iii) abdominal sternites with a distinct dark medial band (Fig. 2M). These characters have already been described and/or depicted in Braasch (1980) and correspond with our observations.

***Electrogena pseudaffinis* (Braasch, 1980)**

The species was described, based on larvae and the original description (Braasch 1980) is basically still the only source that describes its morphology, with some information also being scattered in Sroka and Godunko (2012). *Electrogena pseudaffinis* is distinct from other *Electrogena* species distributed around N. Iraq by: i) shape of pronotum, with posterior corners smoothly rounded, without abrupt step (Fig. 2N); ii) the colour pattern of head and abdominal terga (without distinct median dark band, Fig. 2O); iii) the shape of setae on the dorsal surface of the femora (Fig. 2P, Q); iv) shape of gill plates.

***Caenis luctuosa* (Burmeister, 1839)**

The species is very similar to *C. macrura*, which is widely distributed in the region (being a dominant Caenidae species in neighbouring Iran, see Staniczek et al. 2020). The main distinguishing character is the shape and arrangement of setae on the dorsal surface of the fore-femora. *Caenis luctuosa* possesses a line of short setae, bifurcated to almost half of their length (Fig. 2R), in contrast to the longer setae of *C. macrura*, bifurcated only apically. Both species are widely covered in European determination keys. However, a considerable morphological variability exists within these two species and the identification is not always without doubt. The most detailed description of intraspecific variability in *C. macrura* and *C. luctuosa* was published by Malzacher (1984). In Iraq, there is also the possibility of the occurrence of three more species, described from Israel, namely *Caenis gilbonensis* Malzacher, 1992, *Caenis parabrevipes* Malzacher, 1992 and *Caenis antoniae* Malzacher, 1992 (the latter two species were also reported from Jordan by Gattolliat et al. 2012). Our specimens from Iraq differ from *C. gilbonensis* and *C. parabrevipes* by exhibiting a more deeply and acutely notched posterior margin of sternum X and the presence of more pronounced posterolateral spines on abdominal segments. As for *C. antoniae*, the shield shaped microtrichia on the surface of the larval wing pads are very small in this species (fig. 5e in Malzacher 1992), whereas these structures are much larger in our material identified as *C. luctuosa*.

## Discussion

We listed the records of 37 species for Iraq. From the nine species, we report as new for the country, all of these findings being expected, since these species occur in neighbouring regions and have not been previously mentioned from Iraq only because the area is understudied. The taxonomy of several species we collected is not clear (identified as "cf." in Table 2) . We refrain from providing more precise identifications, since these species require a separate taxonomic revision, based on a more extensive material than specimens merely from Iraq. For *B. lutheri* and *B. vardarensis*, local Caucasian subspecies were described (Zimmermann 1981). However, the delimitation from the nominal subspecies remains questionable, we thus refrain from identification on the subspecies level.

The occurrence of some earlier reported species we consider as highly unlikely and resulting from erroneous identification. This concerns, in particular, the Nearctic species *Baetis tricaudatus* Dodds, 1923 and *Caenis tardata* McDunnough, 1931 (reported in Hanna and Shekha 2015 from the Greater Zab River in Iraqi Kurdistan); *Cinygmula suabequalis* (Banks, 1914) and *Tricothodes albilineatus* Berner, 1946 (reported in Ali and Latef 2017 from the Lesser Zab River in Iraqi Kurdistan) and *Baetis bicaudatus* Dodds, 1923 and *Baetis intercalaris* McDunnough, 1921 (reported in Hanna et al. 2019 from Rawanduz River and the Gali Ali Beg stream in Iraqi Kurdistan). The reports of all these species were most probably caused by the use of American identification keys for Middle Eastern fauna.

A different identification procedure was used by Khudhur and Shekha 2020 for the other three extra-limital mayfly species reported from Iraq, *Epeorus longimanus* Eaton, 1883, *Heptagenia elegantula* (Eaton, 1885) and *Serratella tsuno* Jacobus & McCafferty, 2008 (treated under the name *Ephemerella cornutus* Gose, 1980 - for the classification of this Japanese species, see Jacobus and McCafferty (2008)). In this case, all three species were identified by searching for the most similar 16S sequence in GenBank using BLAST search (Khudhur and Shekha 2020). However, the sequences of a vast majority of Middle Eastern species are not deposited in GenBank; thus, they cannot be associated with a query sequence using BLAST. Moreover, 16S is a relatively conservative gene, so a high degree of similarity does not necessarily imply conspecificity. Therefore, we suggest that all of the abovementioned species no longer be considered as a part of the Iraqi fauna.

The species composition of the area which we sampled in Iraqi Kurdistan is similar to that of the neighbouring mountainous areas of NW Iran and SE Turkey and exhibits some affinities to the Caucasian mayfly fauna. This is evidenced by the occurrence of *B. ilex*, *B. braaschi*, *E. ornatipennis*, *E. pseudaffinis* and *E. nigripilosus*. These represent Caucasian species, sometimes with a territorial extension to the Middle East and Central Asia. On the other hand, some typically Middle Eastern species have also been recorded, such as *B. samochai* (known from Turkey, Israel, Lebanon, Syria and Iran). We have also collected common Palaearctic or west Palaearctic species, some of which have unclear taxonomy and our specimens might actually represent separate Middle East lineages, different from the European ones (B. cf. pentaphlebodes, B. cf. gadeai, C. cf. dipterum).

The future prospects of mayfly research in Iraq include widening the sampling into the southern part of the country, with more affinities to the Levant and Arabian Peninsula. As for the taxa of the mountainous area in the north, it would be useful to include the material from Iraq in more general revisions of Caucasian taxa with complicated taxonomy (such as genera *Baetis* or *Ecdyonurus*).

## Figures and Tables

**Table 1.** List of investigated localities.

**Locality code**	**Specification**	**Latitude (N)**	**Longitude (E)**
IRQ 19/01	IRAQ, Kurdistan, Sulaimaniyah Province, Dukan area, Chami Razan stream, the upstream, leg. Khudhur, 22.1.2019	35°46'36.43"	44°58'29.01"
IRQ 19/02	IRAQ, Kurdistan, Sulaimaniyah Province, Dukan area, Chami Razan stream, the downstream, leg. Khudhur, 22.1.2019	35°48'12.45"	44°58'38.92"
IRQ 19/03	IRAQ, Kurdistan, Erbil Province, Choman Town, Choman Stream, rocky creek behind Halgurd resort, leg. Khudhur, 28.4.2019	36°39'8.29"	44°53'22.34"
IRQ 19/04	IRAQ, Kurdistan, Erbil Province, Choman Town, Warda Village, rocky creek near the village, leg. Khudhur, 29.4.2019	36°40'6.55"	44°52'9.61"
IRQ 19/05	IRAQ, Kurdistan, Erbil Province, Choman Town, Gondazhor Village, creek located NW to the village, leg. Khudhur, 29.4.2019	36°43'20.04"	44°54'56.25"
IRQ 19/06	IRAQ, Kurdistan, Erbil Province, Choman Town, Shewalok Village, creek near the village, leg. Khudhur, 29.4.2019	36°36'0.93"	44°52'31.74"
IRQ 19/07	IRAQ, Kurdistan, Erbil Province, Barzan area, creek near to Shandar cave, leg. Khudhur, 1.5.2019	36°49'16.40"	44°9'53.0"
IRQ 19/08	IRAQ, Kurdistan, Sulaimaniyah Province, Sharbazher area, Upper Dere Village, creek close Gmo Mt. plain, leg. Khudhur, 9.9.2019	35°56'10.48"	45°32'59.35"
IRQ 19/09	IRAQ, Kurdistan, Sulaimaniyah Province, Sharbazner area, Suraze Village, Twlwe Brook, leg. Khudhur, 9.9.2019	35°54'43.12"	45°34'45.28"
IRQ 19/10	IRAQ, Kurdistan, Sulaimaniyah Province, Shavbazner area, Upper Dere Village, Chami Marwe stream, leg. Khudhur, 9.9.2019	35°56'12.36"	45°33'38.76"
IRQ 19/11	IRAQ, Kurdistan, Sulaimaniyah Province, Sharbazher area, Mawat Town, Brook down to the town leg. Khudhur, 13.9.2019	35°53'37.34"	45°24'7.09"
IRQ 19/12	IRAQ, Kurdistan, Sulaimaniyah Province, Hawraman area, Biyara Town, Water Canal East to the town, leg. Khudhur, 14.9.2019	35°13'53.27"	46°7'22.58"E
IRQ 19/13	IRAQ, Kurdistan, Sulaimaniyah Province, Hawraman area, Tawela Town, Awesar stream, leg. Khudhur, 14.9.2019	35°12'53.64"	46°11'13.79"
IRQ 19/14	IRAQ, Kurdistan, Sulaimaniyah Province, BaKrajo area, KaniPan Village, Kandakawa stream, leg. Khudhur, 25.9.2019	35°33'7.2936"	45°58'38.9204"
IRQ 19/15	IRAQ, Kurdistan, Sulaimaniyah Province, Sharbazher area, Qaiwan Village, creek near to the village, leg. Khudhur, 28.9.2019	35°43'40.6427"	45°24'48.3988"
IRQ 19/16	IRAQ, Kurdistan, Sulaimaniyah Province, Sharbazher area, Kuna Masi Village, Kuna Masi stream, the upstream, leg. Khudhur, 28.9.2019	35°47'47.86"	45°24'49.15"
IRQ 19/17	IRAQ, Kurdistan, Sulaimaniyah Province, Sharbazher area, Kuna Masi Village, Kuna Masi stream, the downstream, leg. Khudhur, 28.9.2019	35°47'53.02"	45°24'59.47"
IRQ 19/18	IRAQ, Kurdistan, Erbil Province, Balisan Village, Water Canal of the village leg. Khudhur, 22.10.2019	36°24'14.99"	44°32'52.04"
IRQ 19/19	IRAQ, Kurdistan, Duhok Province, Amedi Town, Solav Resort, Solav waterfall, leg. Khudhur, 23.10.2019	37°6'31.9"	43°28'44.72"
IRQ 19/20	IRAQ, Kurdistan, Duhok Province, Amedi Town,, Anishke cave, brook near to the cave, leg. Khudhur, 23.10.2019	37°6'33.01"	43°21'37.58"
IRQ 19/21	IRAQ, Kurdistan, Duhok Province, Bamarni Town, the water canal, leg. Khudhur, 23.10.2019	37°7'9.84"	43°16'14.68"
IRQ 19/22	IRAQ, Kurdistan, Duhok Province, Bamarni Town, Dihe Village, stream north to the village, leg. Khudhur, 23.10.2019	37°8'26.15"	43°10'40.17"
IRQ 19/23	IRAQ, Kurdistan, Duhok Province, Bamarni Town. KaniBlav, creek in the village leg. Khudhur, 23.10.2019	37°10'33.82"	43°10'43.17"E
IRQ 19/24	IRAQ, Kurdistan, Duhok Province , Amedi Town, Dereluk Stream, leg. Khudhur, 23.10.2019	37°3'4.76"	43°39'42.38"

**Table 2.** List of mayfly species recorded in Iraq.

No.	Species	Numbers of sampled sites	Distribution	Distribution in Iraq	First reference for Iraq
	Baetidae				
1	Baetis (Baetis) buceratus Eaton, 1870	1	West Palaearctic	Duhok, Erbil, Mosul, Sulaymaniyah	Al-Zubaidi and Al-Kayatt (1986)
2	Baetis (Baetis) lutheri Müller-Liebenau, 1967	2, 7, 9, 10, 12, 13, 15, 17-24	Europe, Turkey, Caucasus, Iraq	Duhok, Erbil, Sulaymaniyah	Al-Zubaidi et al. (1987)
3	Baetis (Baetis) cf. pentaphlebodes Ujhelyi, 1966	11, 15, 18, 21, 23	West Palaearctic	Duhok, Erbil, Sulaymaniyah	New record for Iraq
4	Baetis (Baetis) samochai Koch, 1981	14	Turkey, Israel, Lebanon, Iran, Syria	Sulaymaniyah	New record for Iraq
5	Baetis (Baetis) vardarensis Ikonomov, 1962	19, 22	Europe, Caucasus, Iraq	Duhok	Al-Zubaidi and Al-Kayatt (1986)
6	Baetis (Baetis) vernus Curtis, 1834	-	Palaearctic	Erbil	Shekha (2011)
7	Baetis (Rhodobaetis) braaschi Zimmermann, 1980	1, 2, 7, 11, 15, 19-22, 24	Central Asia, Caucasus, Crimea, Turkey, Iran	Duhok, Erbil, Sulaymaniyah	New record for Iraq
8	Baetis (Rhodobaetis) cf. gadeai Thomas, 1999	10	Unclear	Sulaymaniyah	New record for Iraq
9	Baetis (Rhodobaetis) ilex Jacob & Zimmermann, 1978	3, 6, 8-13, 15, 18-23	Caucasus, Iran	Duhok, Erbil, Sulaymaniyah	New record for Iraq
10	Baetis (Rhodobaetis) rhodani (Pictet, 1843)	10, 21	West Palaearctic	Duhok, Erbil, Sulaymaniyah	Al-Zubaidi and Al-Kayatt (1986)
11	Cloeon cf. dipterum (Linnaeus, 1761)	-	Holarctic	Baiji	Carl (1989)
12	*Cloeon simile* Eaton, 1870	-	Palaearctic	Baiji	Carl (1989)
	Isonychiidae				
13	*Isonychia arabica* Al-Zubaidi, Braasch & Al-Kayatt, 1987	-	Iraq	Mosul	Al-Zubaidi et al. (1987))
	Oligoneuriidae				
14	*Oligoneuriella bicaudata* Al-Zubaidi, Braasch & Al-Kayatt, 1987	-	Iraq	Duhok	Al-Zubaidi et al. (1987)
15	*Oligoneuriella tskhomelidzei* Sowa & Zosidze, 1973	-	Caucasus, Turkey, Iraq	Erbil	Al-Zubaidi et al. (1987)
	Heptageniidae				
16	*Anapos kugleri* (Demoulin, 1973)	-	Israel, Iraq, Turkey	Duhok	Al-Zubaidi and Al-Kayatt (1986)
17	Ecdyonurus (Ecdyonurus) ornatipennis Tshernova, 1938	2, 17	Caucasus, Turkey, Iran	Sulaymaniyah	New record for Iraq
18	*Electrogena pseudaffinis* (Braasch, 1980)	8, 9	Caucasus, Turkey, Iran	Sulaymaniyah	New record for Iraq
19	Heptagenia (Heptagenia) sulphurea (Müller, 1776)	-	Palaearctic	Erbil	Shekha (2011)
20	Epeorus (Caucasiron) nigripilosus (Sinitshenkova, 1976)	8, 9	Caucasus, Turkey, Iraq	Erbil, Sulaymaniyah	Al-Zubaidi and Al-Kayatt (1986)
21	Epeorus (Epeorus) zaitzevi Tshernova, 1981	2, 15-17	Caucasus, Turkey, Iran, Iraq	Erbil, Sulaymaniyah	Al-Zubaidi et al. (1987)
22	*Rhithrogena expectata* Braasch, 1979	-	Caucasus, Turkey, Iraq	Erbil	Al-Zubaidi et al. (1987)
23	*Rhithrogena semicolorata* (Curtis, 1834)	-	Europe	Erbil	Shekha (2011)
	Leptophlebiidae				
24	*Choroterpes picteti* (Eaton, 1871)	-	Europe	Baiji	Carl (1989)
25	*Habrophlebia fusca* (Curtis, 1834)	-	West Palaearctic	Erbil	Hanna and Shekha (2015)
	Ephemeridae				
26	Ephemera cf. romantzovi Kluge, 1988	2	Caucasus	Sulaymaniyah	New record for Iraq
	Palingeniidae				
27	*Mortogenesia mesopotamica* (Morton, 1921)	-	Iraq, Iran	Amara	Morton (1921)
	Ephemerellidae				
28	*Ephemerella ignita* (Poda, 1761)	-	Palaearctic	Erbil	Al-Zubaidi et al. (1987)
29	*Torleya major* (Klapálek, 1905)	-	West Palaearctic	Erbil	Al-Zubaidi et al. (1987)
	Caenidae				
30	*Brachycercus harrisella* Curtis, 1834	-	Palaearctic	Erbil	Shekha (2011)
31	*Caenis horaria* (Linnaeus, 1758)	-	Palaearctic	Erbil	Shekha (2011)
32	*Caenis luctuosa* (Burmeister, 1839)	2, 11, 24	Palaearctic	Duhok, Sulaymaniyah	New record for Iraq
33	*Caenis macrura* Stephens, 1836	1, 22	Palaearctic	Duhok, Erbil, Mosul, Sulaymaniyah	Al-Zubaidi et al. (1987)
34	*Caenis pseudorivulorum* Keffermüller, 1960	-	Palaearctic	Baiji	Carl (1989)
35	*Caenis rivulorum* Eaton, 1884	-	Palaearctic	Erbil	Hanna and Shekha (2015)
36	*Caenis robusta* Eaton, 1884	-	Palaearctic	Erbil	Hanna et al. (2019)
	Prosopistomatidae				
37	*Prosopistoma helenae* Bojková & Soldán, 2015	-	Iraq	Mosul	Bojková and Soldán (2015)

**Table 3.** List of extra-limital mayfly species, reported from Iraq, based on misidentifications.

**No.**	**Species**	**Numbers of sampled sites**	**Distribution**	**Reference for Iraq**
1.	*Baetis bicaudatus* Dodds, 1923	-	Nearctic	Hanna et al. (2019)
2.	*Baetis intercalaris* McDunnough, 1921	-	Nearctic	Hanna et al. (2019)
3.	*Baetis tricaudataus* Dodds, 1923	-	Nearctic	Hanna and Shekha (2015)
4.	*Cinygmula subaequalis* (Banks, 1914)	-	Nearctic	Ali and Latef (2017)
5.	*Epeorus longimanus* Eaton, 1883	-	Nearctic	Khudhur and Shekha (2020)
6.	*Heptagenia elegantula* (Eaton, 1885)	-	Nearctic	Khudhur and Shekha (2020)
7.	*Serratella tsuno* Jacobus & McCafferty, 2008		Japan	Khudhur and Shekha (2020)
8.	*Caenis tardata* McDunnough, 1931	-	Nearctic	Hanna and Shekha (2015)
